# Association Between Lower Instrumented Vertebra Selection and Mechanical Complications After Surgical Correction for Kyphotic Deformity Following Osteoporotic Vertebral Fracture

**DOI:** 10.3390/jcm15051731

**Published:** 2026-02-25

**Authors:** Keishi Maruo, Fumihiro Arizumi, Kazuya Kishima, Tetsuto Yamaura, Masaru Hatano, Hayato Oishi, Toshiya Tachibana

**Affiliations:** 1 Department of Orthopaedic Surgery, Hyogo Medical University, 1-1 Mukogawa-cho, Nishinomiya 663-8501, Japan; f_ali_14@yahoo.co.jp (F.A.); kazuya99@live.jp (K.K.); tetsuto.yamaura@icloud.com (T.Y.); m.hatano.108@gmail.com (M.H.); hayatooishi03@gmail.com (H.O.); tachi@hyo-med.ac.jp (T.T.); 2 Department of Orthopaedic Surgery, Miyoshi Hospital, 24-9 Koshien-guchi-kitamachi, Nishinomiya 663-8112, Japan; 3 Department of Orthopaedic Surgery, Daiwa Central Hospital, 1-2-7 Nagahashi, Nishinari-ku, Osaka 557-0025, Japan

**Keywords:** kyphotic deformity following osteoporotic vertebral fracture, corrective surgery, mechanical complications

## Abstract

**Background**: Kyphotic deformity following osteoporotic vertebral fracture (KDOVF) often requires corrective surgery to restore sagittal alignment; however, mechanical complications, such as proximal junctional failure (PJF) and distal junctional failure (DJF), remain major concerns. Selection of the lower instrumented vertebra (LIV) plays a critical role in balancing mechanical stability and functional preservation; however, the optimal criteria for LIV selection have not been fully established. **Methods:** This multicenter retrospective cohort study included 52 patients who underwent corrective surgery for KDOVF, with a minimum 1-year follow-up. The patients were classified into a long-fixation group with pelvic fixation (*n* = 27) and a short-fixation group with lumbar LIV fixation (*n* = 25). Mechanical complications, radiographic parameters, patient-reported outcomes, and paraspinal muscle fatty degeneration were compared between groups. Subgroup analysis was performed within the short-fixation group to identify the factors associated with DJF. **Results:** The incidence of PJF was significantly higher in the long-fixation group than in the short-fixation group (37% vs. 8%, *p* < 0.01), whereas DJF was observed only in the short-fixation group (24%). Within the short-fixation group, patients who developed DJF demonstrated significantly greater preoperative sagittal malalignment, a lower rate of cement-augmented pedicle screw, and more advanced fatty degeneration of the paraspinal muscles. The short-fixation group also showed better postoperative lumbar function. **Conclusions:** LIV selection in KDOVF surgery is associated with distinct patterns of junctional mechanical complications. Short fixation may be feasible in carefully selected patients who demonstrate preserved compensatory capacity.

## 1. Introduction

Kyphotic deformity following osteoporotic vertebral fracture (KDOVF) is a serious condition that increasingly affects the aging population and leads to substantial deterioration of health-related quality of life [1,2,3,4,5,6,7,8]. Progressive sagittal malalignment after vertebral collapse is associated with chronic back pain, impaired mobility, and functional decline [1,3,4,5]. When conservative treatment fails, corrective surgery aimed at restoring sagittal alignment is often required. Although modern corrective techniques, including three column osteotomy or corpectomy, can effectively restore spinal alignment, mechanical complications remain common after surgery for KDOVF [9,10,11,12,13]. Junctional problems, particularly proximal junctional failure (PJF) and distal junctional failure (DJF), are major causes of postoperative morbidity and revision surgery [9,10,12,14]. Fixation extending to the pelvis has been shown to improve global sagittal alignment in patients with severe deformities; however, this approach is associated with increased surgical invasiveness and a higher risk of proximal mechanical complications [9]. In contrast, terminating fixation at the lumbar spine may reduce proximal stress and preserve lumbosacral motion; however, lumbar-lower instrumented vertebra (LIV) constructs are vulnerable to distal junctional failure [10,12]. Recently, the availability of cement-augmented pedicle screws (CAPS) has expanded the surgical options for patients with osteoporotic bone, potentially enhancing the fixation strength in lumbar constructs [15,16]. However, the influence of such advancements on the optimal selection of the LIV in KDOVF surgery has not yet been fully clarified. Therefore, in this study, we aimed to evaluate the surgical outcomes after corrective surgery for KDOVF, focusing on the association between LIV selection and mechanical complications.

## 2. Materials and Methods

### 2.1. Study Design and Patient Population

In this multicenter retrospective cohort study, we reviewed consecutive patients who underwent spinal correction for KDOVF at our institution between January 2019 and December 2024. KDOVF was defined as a symptomatic kyphotic deformity that developed after OVF and required surgical correction because of progressive deformity, pain, or functional impairment. The inclusion criteria were as follows: (1) diagnosis of KDOVF, (2) surgical treatment performed during the study period, and (3) availability of at least 1 year of postoperative follow-up. Patients were excluded if they had (1) a history of spinal fusion surgery, (2) active or previous spinal infection, or (3) severe neurological deficits that could significantly affect postoperative functional evaluation. Fifty-two patients met the inclusion criteria and were enrolled in this study. Parkinson’s disease was present in two patients in the long fixation group and one patient in the short fixation group. Clinical, radiographic, and patient-reported outcome data were retrospectively obtained from the electronic medical records system of our institution. This study was conducted in accordance with the principles of the Declaration of Helsinki and approved by the institutional review board of our institution.

### 2.2. Surgical Strategy and Group Classification

Patients were classified into two groups according to the LIV. Those who required fixation extending to the pelvis were assigned to the long-fixation group (*n* = 27), whereas those whose LIV was located in the lumbar spine were assigned to the short-fixation group (*n* = 25). The LIV was selected by the operating surgeon based on individual patient factors, including deformity severity, bone quality, and sagittal spinal alignment. All cases were instrumented using polyaxial pedicle screws with 5.5-mm titanium rods. Since 2023, CAPS have been selectively used in the short group to enhance the fixation strength in osteoporotic bones.

### 2.3. Outcome Measures

Mechanical complications, including PJF, DJF, and rod fractures, were evaluated. PJF was defined as follows: (1) fracture of the upper instrumented vertebra (UIV) or UIV + 1; (2) failure of fixation at UIV; and (3) need for revision surgery due to PJF [17]. DJF was defined as follows: (1) fracture of the LIV or LIV-1; (2) failure of fixation at the LIV; and (3) need for revision surgery due to DJF. Radiographic assessments were performed using standing whole-spine lateral radiographs obtained preoperatively, immediately postoperatively, and at the final follow-up visit. The following radiographic parameters were measured: thoracic kyphosis (TK), thoracolumbar kyphosis (TLK), lumbar lordosis (LL), lower lumbar lordosis (LLL), pelvic incidence minus lumbar lordosis mismatch (PI–LL), and sagittal vertical axis (SVA). Patient-reported outcome measures (PROMs) were assessed using the Japanese Orthopaedic Association Back Pain Evaluation Questionnaire (JOABPEQ), visual analog scale (VAS) for low back pain (LBP), and Oswestry Disability Index (ODI).

### 2.4. Subgroup Analysis

For subgroup analysis, patients in the short group were further divided into the DJF and non-DJF groups. Fatty degeneration of the paraspinal muscles was evaluated on preoperative axial T1-weighted MRI at L4-L5 and categorized into five stages according to the Goutallier classification [1,18]: stage 0, no fatty degeneration; stage 1, minimal fatty degeneration; stage 2, more muscle than fat; stage 3, equal muscle and fat composition; and stage 4, less muscle than fat. Radiographic parameters and use of CAPS were compared between the two groups to evaluate potential factors associated with DJF.

### 2.5. Statistical Analysis

Continuous variables are presented as the mean ± standard deviation, and categorical variables are expressed as counts and percentages. Comparisons between the long and short-fixation groups were performed using Student’s t-test or Mann–Whitney U test for continuous variables, as appropriate, based on data distribution. Categorical variables were compared using the chi-squared test or Fisher’s exact test. All statistical analyses were performed using JMP software (version 19; SAS Institute Inc., Cary, NC, USA). Statistical significance was set at a *p*-value < 0.05.

## 3. Results

### 3.1. Baseline Characteristics

**Figure 1:**
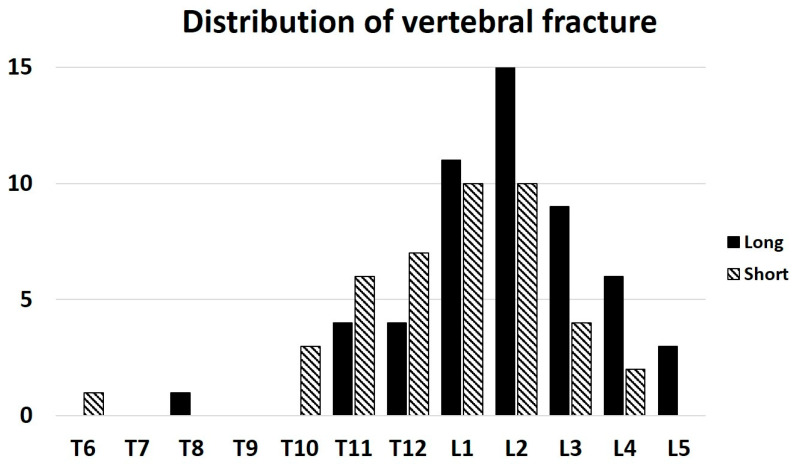
Distribution of osteoporotic vertebral fracture levels by fixation group.

The long- and short-term groups showed no significant differences in age, sex, body mass index, or follow-up period (Table 1). The bone mineral density, expressed as the percentage of the young adult mean at the total hip (YAM [TH]), was significantly lower in the short-fixation group compared with the long-fixation group (67.8% vs. 76.4%, *p* < 0.01). Regarding the fracture characteristics and spinopelvic alignment, OVFs at or below L4 were significantly more frequent in the long-fixation group than in the short-fixation group (26% vs. 4%, *p* = 0.03). In the long fixation group, OVFs were most frequently located at L2 (*n* = 15) and L1 (*n* = 11), followed by L3 (*n* = 9), L4 (*n* = 6), T11 (*n* = 4), T12 (*n* = 4), and T8 (*n* = 1). No OVFs were observed at T6, T7, T9, or T10. In the short-fixation group, OVFs were more cranially distributed, most commonly at L1 and L2 (each *n* = 10), followed by T12 (*n* = 7), T11 (*n* = 6), T10 (*n* = 3), L3 (*n* = 4), L4 (*n* = 2), and T6 (*n* = 1), with no cases at T7, T8, T9, or L5 (Figure 1).

### 3.2. Osteoporosis Medications and Surgical Procedures

The use of medications for osteoporosis, including antiresorptive and anabolic agents, did not differ significantly between the long and short-fixation groups (Table 2). In the short-fixation group, the LIV was distributed from L1 to L5, with L3 being the most common level (L1: *n* = 2, L2: *n* = 5, L3: *n* = 9, L4: *n* = 5, and L5: *n* = 4). In contrast, all patients in the long-fixation group underwent fixation extending to the pelvis (*n* = 25). The surgical approach was selected according to the severity of the vertebral collapse, endplate damage, and rigidity of the kyphotic deformity, with either anterior or posterior osteotomy used as appropriate. With respect to surgical techniques, the distribution of surgical approaches (anterior vs. posterior osteotomy–based correction) and the use of osteotomy did not differ significantly between the long and short-fixation groups.

### 3.3. Mechanical Complications

The incidence of PJF was significantly higher in the long-fixation group than in the short-fixation group (37% vs. 8%, *p* < 0.01) (Table 3). In contrast, DJF occurred exclusively in the short-fixation group, with an incidence of 24%, whereas no cases of DJF were observed in the long-fixation group (*p* < 0.01). Furthermore, revision surgery due to DJF was required in 16% of patients in the short-fixation group, whereas no revision surgeries for DJF were performed in the long-fixation group (*p* = 0.03). Although rod fractures occurred more frequently in the long-fixation group compared with the short-fixation group (18.5% vs. 4%), the difference was not statistically significant (*p* = 0.10).

### 3.4. Radiographic Parameters

Preoperatively, the long-fixation group demonstrated significantly more severe sagittal malalignment than the short-fixation group (Table 4). TK and TLK were significantly smaller in the long-fixation group than in the short-fixation group (TK: 18.3 ± 17.8° vs. 31.4 ± 19.8°, *p* = 0.02; TLK: 29.7 ± 24.2° vs. 42.4 ± 20.7°, *p* = 0.049). LL and LLL were markedly reduced in the long-fixation group (LL: 4.2 ± 17.5° vs. 24.9 ± 21.6°, *p* < 0.01; LLL: 18.2 ± 13.5° vs. 32.0 ± 12.6°, *p* < 0.01). Consequently, the long-fixation group exhibited a significantly larger PI–LL mismatch (44.4 ± 20.0° vs. 20.8 ± 21.1°, *p* < 0.01), greater PT (38.6 ± 11.7° vs. 30.8 ± 8.4°, *p* < 0.01), and increased SVA (137.4 ± 54.2 mm vs. 81.7 ± 49.0 mm, *p* < 0.01).

### 3.5. Postoperative Radiographic Parameters

Postoperatively, both groups showed substantial improvement in sagittal alignment. LL was significantly greater in the long-fixation group than in the short-fixation group (45.0 ± 10.1° vs. 34.8 ± 7.3°, *p* < 0.01). The PI–LL mismatch was also significantly smaller in the long-fixation group (3.6 ± 10.3° vs. 11.0 ± 9.2°, *p* < 0.01), indicating superior global sagittal correction.

### 3.6. Subgroup Analysis in the Short-Fixation Group

Subgroup analysis was performed within the short-fixation group by comparing patients who developed DJF with those who did not (non-DJF) (Table 5). Preoperative SVA was significantly higher in the DJF group than in the non-DJF group (109.3 ± 35.2 mm vs. 49.2 ± 40.4 mm, *p* = 0.02), while the prevalence of CAPS usage was significantly lower in the DJF group (16.7% vs. 63.0%, *p* = 0.046). The severity of fatty degeneration of the paraspinal muscles was significantly higher in the DJF group than in the non-DJF group (*p* = 0.02).

### 3.7. Patient-Reported Outcome Measures (PROMs)

The PROMs at baseline and final follow-up are summarized in Table 6. The long- and short-fixation groups showed no significant difference in JOABPEQ domains, including pain-related disorders, walking ability, social life function, and mental health. In contrast, the short-fixation group demonstrated significantly better lumbar function and significantly lower ODI scores than the long-fixation group at final follow-up.

### 3.8. Representative Case

Case 1: Long-fixation case

**Figure 2:**
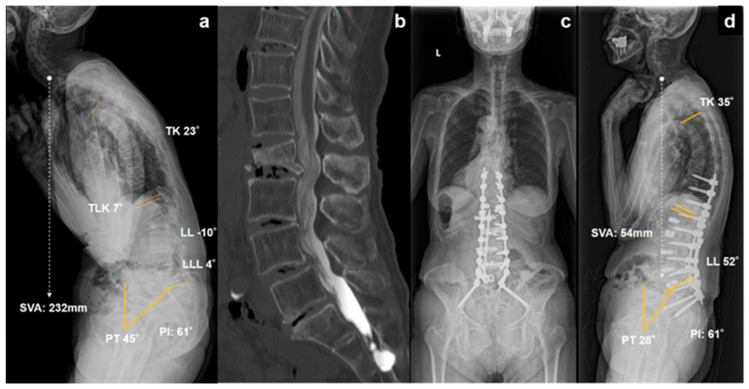
Representative case of long-fixation with pelvic fixation (Case 1). Preoperative standing lateral radiograph showing severe kyphotic deformity following an osteoporotic vertebral fracture (OVF) at L2 with decompensated sagittal malalignment (SVA 232 mm, LL −10°, LLL 4°) (**a**). Computed tomography demonstrated advanced degenerative changes in lower lumbar intervertebral discs (**b**). Postoperative anteroposterior (**c**) and lateral (**d**) standing radiographs after staged lateral lumbar interbody fusion and posterior long-segment fixation from T10 to the pelvis demonstrate satisfactory correction of global sagittal alignment.

A 77-year-old woman presented with a severe kyphotic deformity following an OVF at L2 (Figure 2a). The patient demonstrated decompensated severe sagittal malalignment (SVA 232 mm), with a LL of −10° and LLL of 4°. Computed tomography (CT) revealed degenerative changes in the lower lumbar intervertebral discs (Figure 2b). The patient underwent staged surgery with lateral lumbar interbody fusion and posterior long-segment fixation from T10 to the pelvis. Satisfactory correction of the global sagittal alignment was achieved after surgery (Figure 2c,d).

Case 2: DJF requiring extension to the pelvis (Short-fixation group without CAPS)

**Figure 3:**
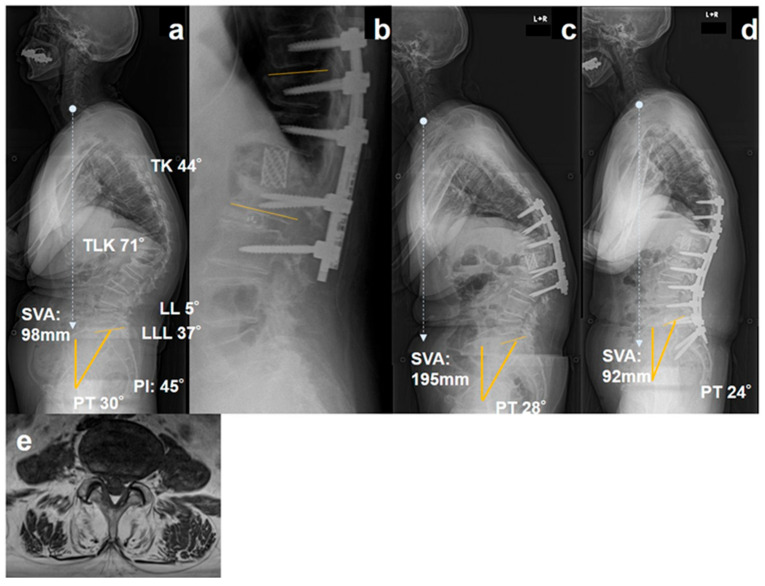
Distal junctional failure after short fixation without CAPS (Case 2). Preoperative standing lateral radiograph of a patient with an old OVFs from T10 to L2 showing moderate sagittal malalignment (SVA, 98 mm; PT, 30°; LL, 5°; LLL, 37°) (**a**). Postoperative lateral radiograph after posterior vertebral column resection and pedicle screw fixation showing acceptable local correction (**b**). Early distal junctional failure developed within 2 months after surgery with progressive collapse at the distal junction (**c**). Revision surgery with extension of fixation to the pelvis is required to restore sagittal balance (**d**). Axial T1-weighted MRI at the L4–L5 shows paraspinal muscle fatty degeneration corresponding to Goutallier grade 3 (**e**).

A 72-year-old woman presented with an OVF from T10 to L2 (Figure 3a). The patient demonstrated a moderate sagittal malalignment (PT, 30°; SVA, 98 mm) with a LL of 5° and a LLL of 37°. The patient underwent a posterior vertebral column resection with pedicle screw fixation (Figure 3b). Postoperative radiographs demonstrated acceptable local correction; however, DJF developed within 2 months postoperatively (Figure 3c). Progressive collapse and sagittal imbalance led to worsening of symptoms, and revision surgery with extension of the fixation to the pelvis was required (Figure 3d). MRI showed Goutallier grade 3 paraspinal muscle fatty degeneration (Figure 3e).

Case 3: Lumbar fixation with CAPS without revision surgery (short group with CAPS)

**Figure 4:**
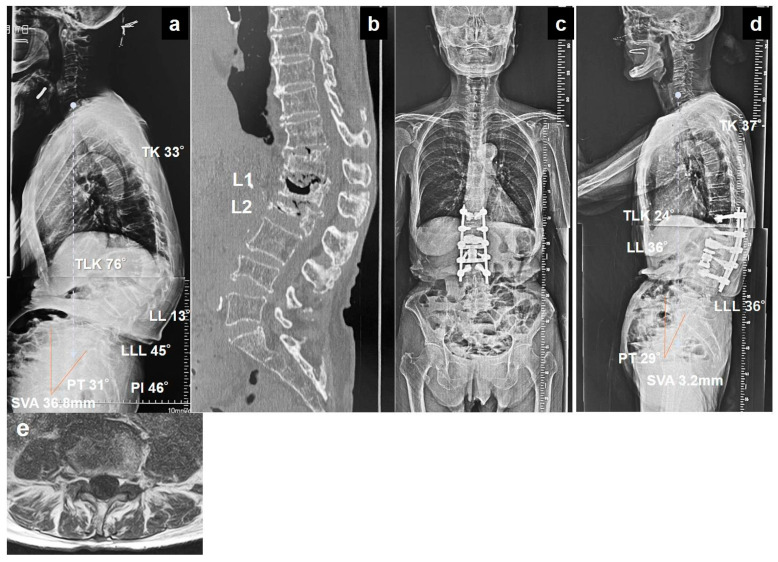
Short fixation with CAPS without revision surgery (Case 3). Preoperative standing lateral radiograph (**a**) and CT (**b**) demonstrating kyphotic deformity due to severe wedge deformity at L2 and nonunion at L1. The patient maintained compensated global sagittal alignment (SVA, 36.8 mm) with pelvic retroversion (PT, 31°) and lower lumbar lordosis (45°). Postoperative radiographs after staged kyphoplasty, L1 corpectomy, and posterior percutaneous pedicle screw fixation using cement-augmented pedicle screws demonstrate restoration of satisfactory sagittal alignment without the need for pelvic fixation (**c**,**d**). Axial T1-weighted MRI at the L4–L5 demonstrates mild fatty degeneration of the paraspinal muscles, corresponding to Goutallier grade 2 (**e**).

An 83-year-old woman with a KDOVF was diagnosed with severe wedge deformity at L2 and nonunion at L1 (Figure 4a,b). The patient demonstrated compensated sagittal global alignment (SVA, 36.8 mm), pelvic retroversion (PT, 31°), and a LLL of 45°. She underwent staged kyphoplasty and posterior PPS fixation using CAPS and corpectomy at L1. Postoperative radiographs demonstrated restoration of satisfactory sagittal alignment (Figure 4c,d). MRI showed Goutallier grade 2 paraspinal muscle fatty degeneration (Figure 4e).

## 4. Discussion

In the present study, we observed that the selection of the LIV in corrective surgery for KDOVF was associated with distinct patterns of proximal and distal junctional mechanical complications. Long-fixation extending to the pelvis achieved superior postoperative sagittal alignment, as evidenced by a significantly greater LL and smaller PI–LL mismatch; however, it was associated with a markedly higher incidence of PJF. In contrast, short fixation substantially reduced PJF but predisposed patients to DJF, which occurred exclusively in this group. These findings are consistent with previous reports indicating that extensive spinopelvic fixation improves global alignment at the cost of increased proximal mechanical stress, whereas lumbar LIV constructs preserve mobility, but are vulnerable to distal breakdown [9,10,12]. Importantly, our subgroup analysis suggests that the selective use of CAPS may mitigate the risk of DJF in short-fixation constructs, highlighting a potential strategy to balance invasiveness and mechanical durability. Importantly, the PROMs revealed different perspectives on functional outcomes. Despite inferior global sagittal correction, the short-fixation group demonstrated significantly better lumbar function on the JOABPEQ and a lower ODI. These findings suggest that the preservation of lumbar mobility may contribute to superior functional outcomes when excessive sagittal imbalance is absent. Therefore, in patients without severe preoperative sagittal malalignment, fixation limited to the lumbar spine may be the preferred strategy that balances mechanical safety with functional preservation. In particular, the limited sample size, especially in the subgroup analyses, may have reduced the ability to detect small but clinically relevant associations and should be considered when interpreting the negative findings.

However, these results should be interpreted with caution, as patients selected for short fixation generally exhibited less severe preoperative sagittal malalignment and greater compensatory capacity. In this context, preserved mobility of the unfused lumbar segments may have contributed to favorable functional outcomes despite residual sagittal imbalance. Therefore, short fixation should not be considered a superior strategy in itself, but it may be a feasible option for carefully selected patients without significant preoperative sagittal decompensation. Recent advancements, such as CAPS, may further enhance the durability of lumbar-ending constructs and help mitigate the risk of DJF, thereby expanding the indications for short-fixation in selected patients with KDOVF.

KDOVF differs fundamentally from post-traumatic deformities in younger patients, as it is characterized by severe bone fragility, progressive vertebral collapse, and impaired compensatory mechanisms. Consequently, corrective surgery for KDOVF often requires aggressive techniques, such as three-column osteotomy [11], vertebral column resection [19], or anterior column reconstruction to restore sagittal balance [13]. Previous studies have demonstrated that long-segment fixation, with or without pelvic fixation, can effectively correct rigid kyphosis; however, this strategy is associated with a high incidence of junctional complications, particularly in elderly patients with compromised bone quality [9,10,12]. In contrast, recent reports suggest that carefully selected short-segment or lumbar LIV constructs may provide acceptable deformity correction in patients with preserved compensatory capacity, especially when fixation strength is augmented using bone cement [20]. Notably, Zhang et al. emphasized that osteoporosis itself represents the fundamental pathology underlying OVF-related deformities and that surgical correction alone is insufficient without continuous pharmacological management. Our findings are consistent with this concept and demonstrate that short-fixation can be a viable surgical option in selected patients with KDOVF, provided that the preoperative sagittal malalignment is not excessive and the fixation strength is adequately reinforced. Furthermore, our results suggest that careful selection of the LIV, supported by CAPS and optimized osteoporosis treatment, may allow short fixation to serve as a feasible alternative to pelvic fixation, thereby reducing surgical invasiveness while maintaining acceptable mechanical stability. Although total hip BMD was significantly lower in the short-fixation group, vertebral bone quality was comparable between groups. Preoperative osteoporosis medications were not administered for a sufficiently long duration to produce distinct site-specific effects on vertebral versus femoral bone, and baseline patient characteristics were largely similar. Therefore, the underlying reason for this discrepancy could not be clearly identified and should be interpreted with caution.

Recent evidence has increasingly emphasized the critical role of the fracture location in determining sagittal alignment deterioration following OVFs [4,21,22]. Yokoyama et al. demonstrated that a newly developed OVF resulted in an average increase in SVA of 2.8 cm, with fractures at or below L1 carrying a significantly greater risk of sagittal imbalance than thoracic fractures [21]. Similarly, Plais et al. reported that lumbar OVFs and multiple fractures involving the thoracolumbar or lumbar regions were strong risk factors for sagittal malalignment in elderly patients [22]. Our recent multicenter analysis further supports these findings by demonstrating that OVFs localized to the lower lumbar spine (L3–L5) are strongly associated with severe global sagittal malalignment, reduced LLL, and inferior functional outcomes, including lower JOABPEQ gait function scores and higher ODI values [4]. Recent studies have shown that distal junctional complications are strongly influenced by the structural integrity and compensatory capacity of the lower lumbar spine. Sawada et al. demonstrated that distal junctional kyphosis frequently occurs when the stable sagittal vertebra (SSV) is excluded from the fixation range, particularly in the presence of instability at the distal adjacent disc [10]. Kudo et al. reported a high incidence of DJF after major corrective surgery without pelvic fixation, with failure strongly associated with preoperative sagittal malalignment (SVA > 80 mm), reduced LL and LLL, increased PT (PT > 28°), and PI–LL mismatch [12]. In the present study, sensitivity analyses such as ROC modeling within the short-fixation group were not performed because of the limited number of DJF events, which would have compromised statistical reliability. Nevertheless, when our cohort was interpreted in light of previously reported alignment thresholds, patients selected for lumbar LIV fixation generally exhibited milder preoperative sagittal malalignment and preserved compensatory capacity of the lower lumbar spine. Based on these findings, shortening the fusion levels with the lumbar LIV may be a reasonable option in carefully selected patients with KDOVF. Specifically, patients with pelvic tilt < 30°, SVA < 100 mm, LLL > 30°, and no involvement of severe lower lumbar OVFs demonstrated sufficient residual compensatory capacity to maintain postoperative sagittal balance. In these cases, augmentation with CAPS appeared to enhance distal foundation stability, potentially reducing the risk of DJF. These criteria are consistent with the previously reported risk thresholds for DJF and emphasize the importance of preserving the lordotic foundation and mechanical integrity of the lower lumbar spine. Although pelvic fixation remains necessary in patients with impaired compensation or lower lumbar OVF, our results suggest that, under these conditions, short fixation may be feasible without compromising mechanical stability.

**Figure 5:**
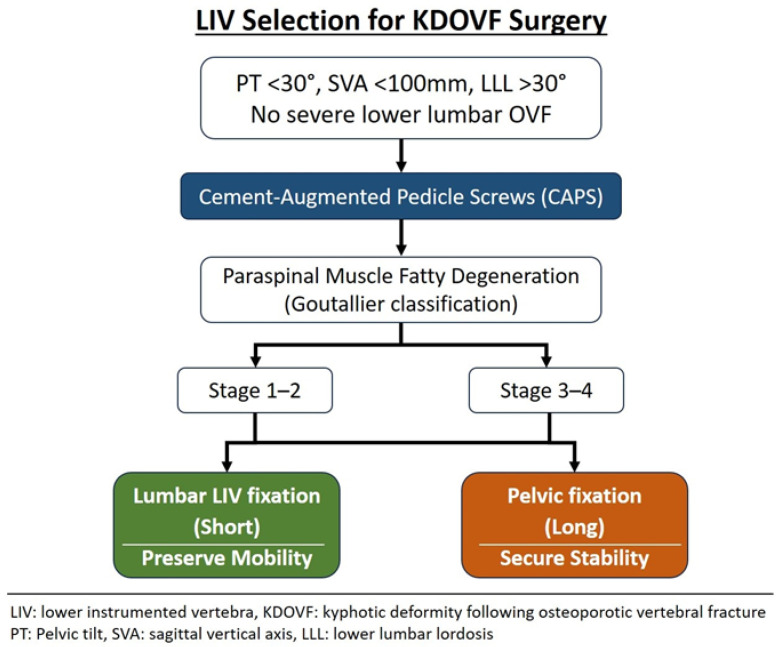
Conceptual framework illustrating clinical considerations for LIV selection in KDOVF.

Recent evidence has highlighted paraspinal muscle degeneration as a critical biological factor that contributes to the progression of kyphotic deformity and mechanical instability following OVF [1,2,23]. Li et al. demonstrated that severe fatty degeneration of the multifidus muscle was significantly associated with sagittal malalignment, loss of correction, and mechanical complications after surgery for OVF-related kyphosis [23]. Similarly, Seo et al. reported that fatty degeneration of the multifidus and erector spinae muscles was strongly associated with progressive segmental kyphosis after conservative treatment, suggesting that impaired muscle quality promotes local kyphotic progression even in the absence of surgical intervention [2]. Kusukawa et al. identified severe paraspinal muscle fatty degeneration as an independent risk factor for domino OVFs [1]. Our results demonstrate that more severe fatty degeneration of the paraspinal muscles is associated with the incidence of DJF. These findings have important implications for LIV selection during KDOVF. When fixation stops at the lumbar spine, the remaining non-fused lumbar segments are required to function as dynamic stabilizers to maintain global sagittal alignment. Therefore, in addition to fracture location, spinopelvic alignment, and bone quality, careful assessment of fatty degeneration in the paraspinal muscles at the unfused lumbar levels is essential. Based on these findings, we present a conceptual framework to illustrate clinical factors associated with LIV selection in corrective surgery for KDOVF (Figure 5), rather than a prescriptive indication algorithm. In patients without marked preoperative sagittal malalignment (PT < 30°, SVA < 100 mm, and LLL > 30°) and without severe lower lumbar OVF, short fixation may be feasible, reflecting preserved compensatory capacity rather than a definitive indication. In such cases, the use of CAPS may enhance distal fixation strength. The degree of paraspinal muscle fatty degeneration, assessed using the Goutallier classification, is incorporated as an additional contextual factor when considering the fixation range: patients with mild degeneration (stages 1–2) were more frequently treated with short fixation in this cohort, whereas those with advanced degeneration (stages 3–4) more often required pelvic fixation to achieve mechanical stability. This framework is intended to summarize observed clinical patterns and should be interpreted as hypothesis-generating rather than as a guideline for surgical indication.

Conceptual framework summarizing clinical factors associated with LIV selection in KDOVF surgery, including sagittal alignment parameters, the use of cement-augmented pedicle screws (CAPS), and paraspinal muscle fatty degeneration. This figure reflects observed patterns in the present cohort and is intended to illustrate feasibility considerations rather than to define surgical indications.

## 5. Limitations

This study has several limitations. First, its retrospective design and relatively small sample size may have introduced selection bias and limited the statistical power to detect all potential risk factors for mechanical complications. In particular, the limited sample size, especially in the subgroup analyses, may have reduced the ability to detect small but clinically relevant associations and should be considered when interpreting the negative findings. Second, the selection of the LIV was not standardized and was determined by the operating surgeon based on clinical judgment, which may have influenced group allocation. Accordingly, the observed differences in mechanical complications should be interpreted in the context of baseline disease severity that guided LIV selection, and not as a direct comparison of treatment efficacy between fixation strategies. Third, CAPS were selectively introduced during the later study period based on surgeon judgment, primarily in cases with poor bone quality or concern for distal fixation strength. As CAPS use was non-randomized and temporally clustered, the potential influence of a learning-curve or time-related bias on DJF incidence cannot be completely excluded. Finally, a longer follow-up period is required to determine whether the observed differences in mechanical complications and functional outcomes persist in the mid- to long-term period. Despite these limitations, the consistent association among LIV selection, sagittal alignment, CAPS use, and paraspinal muscle degeneration provides meaningful insights into surgical decision-making for KDOVF.

## 6. Conclusions

Corrective surgery for KDOVF is associated with different patterns of proximal and distal junctional mechanical complications depending on the selection of the LIV. Pelvic fixation provides reliable sagittal correction, but increases the risk of PJF, whereas short fixation preserves lumbar mobility, but predisposes patients to DJF. Our results indicate that short fixation may be feasible in carefully selected patients without severe preoperative sagittal malalignment and lower lumbar OVF who demonstrate preserved compensatory capacity, particularly when CAPS are used. Importantly, severe fatty degeneration of the paraspinal muscles at the unfused lumbar levels is associated with DJF and represents an additional biological risk factor. Incorporating muscle quality into the preoperative assessment may refine LIV selection and improve the mechanical durability and functional outcomes of KDOVF surgery.

## Figures and Tables

**Table 1:** Baseline characteristics.

	Long Group	Short Group	*p*-Value
	n (%)	n (%)	
Age (y)	76 ± 4.8	73.4 ± 7.5	0.14
Sex (female)	21 (78)	22 (89)	0.3
BMI	21.3 ± 2.8	21.4 ± 3.9	0.93
YAM (%)			
Lumbar	78.5 ± 16.6	76.2 ± 12.5	0.62
Total hip	76.4 ± 11.1	67.5 ± 7.8	<0.01 *
Multiple OVF	14 (51.8)	10 (40)	0.39
Lower lumbar OVF (L4, L5)	7 (26)	1 (4)	0.03 *
Follow-up period (months)	39.7 ± 21.2	42.3 ± 24.1	0.68

BMI: Body mass index, YAM: Young Adult Mean, OVF: Osteoporotic vertebral fracture, * Statistically significant.

**Table 2:** Types of Anti-osteoporosis medications and surgical procedures.

	Long Group	Short Group	*p*-Value
Anti-osteoporosis medications (n, [%])			0.11
Teriparatide	20 (74)	11 (44)	
Romosozumab	5 (18.5)	12 (48)	
Abaloparatide	1 (3.7)	0	
Bisphosphonate	0	1 (4)	
None	1 (3.7)	1 (4)	
UIV (n)			0.52
T2	1	0	
T5	1	2	
T8	0	1	
T9	6	5	
T10	18	11	
T11	0	6	
Type of surgery (n, [%])			0.42
Corpectomy or LIF + PSF	19 (70.4)	14 (56)	
PVCR	6 (22)	7 (28)	
PSO	2 (7.4)	2 (8)	
VP + PSF	0	2 (8)	

UIV: Upper instrumented vertebra, LIV: Lower instrumented vertebra, LIF: Lateral lumbar interbody fusion, PSF: Posterior spinal fusion, PVCR: Posterior vertebral column resection, PSO: Pedicle subtraction osteotomy, VP: Vertebroplasty.

**Table 3:** Incidence of mechanical complications.

	Long Group	Short Group	*p*-Value
PJF [n, (%)]	10 (37)	2 (8)	<0.01 *
UIV fracture (n)	9	2	
Failure of fixation (n)	1	0	
Revision PJF [n, (%)]	3 (11)	0	0.08
RF [n, (%)]	5 (18.5)	1 (4)	0.1
Revision RF [n, (%)]	3 (11)	1 (4)	0.33
DJF [n, (%)]	0	6 (24)	<0.01 *
LIV fracture (n)	0	4	
failure of fixation (n)	0	2	
Revision-DJF [n, (%)]	0	4 (16)	0.03 *

PJF: Proximal junctional failure, RF: Rod fracture, DJF: Distal junctional failure. UIV: upper instrumented vertebra, LIV: lower instrumented vertebra, * Statistically significant.

**Table 4:** Preoperative and immediate postoperative sagittal spinal alignment.

Spinal Sagittal Alignment		Long Group	Short Group	*p*-Value
Preoperative	TK (°)	18.3 ± 17.8	31.4 ± 19.8	0.02 *
	TLK (°)	29.7 ± 24.2	42.4 ± 20.7	0.049 *
	LL (°)	4.2 ± 17.5	24.9 ± 21.6	<0.01 *
	LLL (°)	18.2 ± 13.5	32 ± 12.6	<0.01 *
	PI-LL (°)	44.4 ± 20	20.8 ± 21.1	<0.01 *
	PT (°)	38.6 ± 11.7	30.8 ± 8.4	<0.01 *
	SVA (mm)	137.4 ± 54.2	81.7 ± 49	<0.01 *
Postoperative (immediately)	TK (°)	35.2 ± 9.7	31 ± 10.4	0.13
	TLK (°)	15.3 ± 6.7	15.9 ± 9.2	0.79
	LL (°)	45 ± 10.1	34.8 ± 7.3	<0.01 *
	LLL (°)	26.3 ± 6	27.4 ± 8.1	0.60
	PI-LL (°)	3.6 ± 10.3	11 ± 9.2	<0.01 *
	PT (°)	23.9 ± 9.2	25.7 ± 7.8	0.46
	SVA (mm)	29.9 ± 45.3	42.7 ± 33.6	0.25

OVF: Osteoporotic vertebral fracture, TK: Thoracic kyphosis, TLK: Thoracolumbar kyphosis. LL: Lumbar lordosis, LLL: Lower lumbar lordosis, PI: Pelvic incidence, PT: Pelvic tilt, SVA: Sagittal vertical axis, * Statistically significant.

**Table 5:** Comparison of preoperative radiographic parameters and use of CAPS between the DJF and non-DJF groups.

Variable	Non-DJF Group	DJF Group	*p*-Value
	(*n* = 19)	(*n* = 6)	
TK (°)	31.6 ± 16.8	41.5 ± 19.7	0.24
TLK (°)	39.9 ± 21.8	50.3 ± 26.9	0.38
LL (°)	26.6 ± 23	27.7 ± 25.6	0.92
LLL (°)	29.5 ± 14.6	32 ± 13.5	0.71
PI-LL (°)	27.4 ± 24	20.8 ± 25.4	0.56
PT (°)	28.3 ± 9.7	34.2 ± 10.1	0.21
SVA (mm)	49.2 ± 40.4	109.3 ± 35.2	0.02 *
Use of CAPS [n, (%)]	12 (63)	1 (16.7)	0.046 *
Goutallie stage			0.02 *
2	11	0	
3	7	4	
4	1	2	
YAM (%)			
Lumbar	78.5 ± 16.6	76.2 ± 12.5	0.62
Total hip	76.4 ± 11.1	67.5 ± 7.8	<0.01 *
Medications (n)			0.82
Teriparatide	6	5	
Romosozumab	11	1	
Bisphosphonate	1	0	
None	1	0	

CAPS: Cement-augmented pedicle screws, DJF: Distal junctional failure, TK: Thoracic kyphosis, TLK: Thoracolumbar kyphosis, LL: Lumbar lordosis, LLL: Lower lumbar lordosis, PI: Pelvic incidence, PT: Pelvic tilt, SVA: Sagittal vertical axis, YAM: Young Adult Mean, * Statistically significant.

**Table 6:** Comparison of patient-reported outcomes between the long group and the short group.

	Long Group	Short Group	*p*-Value
JOABPEQ			
Pain-related disorder			
Baseline	45.9 ± 40.3	18.4 ± 19.6	0.16
Final follow-up	75.6 ± 22.7	71.4 ± 3.6	0.63
Lumbar function			
Baseline	49.1 ± 25.9	24.2 ± 17.1	0.1
Final follow-up	43.6 ± 22.7	75.6 ± 5.4	<0.01 *
Walling ability			
Baseline	21.8 ± 28	20 ± 17.2	0.89
Final follow-up	53.7 ± 23.1	60 ± 12.6	0.57
Social life function			
Baseline	39.1 ± 18.8	31.4 ± 20.1	0.43
Final follow-up	45.5 ± 21.3	56.2 ± 21.6	0.26
Mental health			
Baseline	33.8 ± 18	46.4 ± 15.8	0.17
Final follow-up	47.8 ± 17.5	57.6 ± 13.6	0.26
VAS for low back pain			
Baseline	70.9 ± 19.9	77.8 ± 16.2	0.46
Final follow-up	39.2 ± 25.8	21 ± 10.7	0.11
ODI			
Baseline	50 ± 10.9	51.7 ± 17.5	0.76
Final follow-up	33 ± 14	19.5 ± 9.8	0.04 *

JOABPEQ: Japanese Orthopedic Association Back Pain Evaluation Questionnaire, VAS: Visual analog scale, ODI: Oswestry Disability Index. * Statistically significant.

## Data Availability

The datasets used and/or analyzed in the current study are available from the corresponding author upon reasonable request.

## Abbreviations

The following abbreviations are used in this manuscript:

BMD	Bone mineral density
BMI	Body mass index
CAPS	Cement-augmented pedicle screws
CT	Computed tomography
DJF	Distal junctional failure
GSA	Global sagittal alignment
JOABPEQ	Japanese Orthopaedic Association Back Pain Evaluation Questionnaire
KDOVF	Kyphotic deformity following osteoporotic vertebral fracture
LIV	Lower instrumented vertebra
LL	Lumbar lordosis
LLL	Lower lumbar lordosis
MRI	Magnetic resonance imaging
ODI	Oswestry Disability Index
OVF	Osteoporotic vertebral fracture
PI	Pelvic incidence
PJF	Proximal junctional failure
PT	Pelvic tilt
SVA	Sagittal vertical axis
TK	Thoracic kyphosis
TL	Thoracolumbar
TLK	Thoracolumbar kyphosis
UIV	Upper instrumented vertebra
YAM	Young adult mean
